# Corrupted Point Cloud Classification Through Deep Learning with Local Feature Descriptor

**DOI:** 10.3390/s24237749

**Published:** 2024-12-04

**Authors:** Xian Wu, Xueyi Guo, Hang Peng, Bin Su, Sabbir Ahamod, Fenglin Han

**Affiliations:** 1 School of Mechanical and Electrical Engineering, Central South University, Changsha 410083, China; wuxian@csu.edu.cn (X.W.); penghdiyu@csu.edu.cn (H.P.); 223711075@csu.edu.cn (B.S.); ahamodx@gmail.com (S.A.); 2 Resource Recycling Research Institute, Central South University, Changsha 410083, China; xyguo@csu.edu.cn

**Keywords:** deep neural networks, local feature descriptor, object classification, point cloud, partial point cloud

## Abstract

Three-dimensional point cloud recognition is a very fundamental work in fields such as autonomous driving and face recognition. However, in real industrial scenarios, input point cloud data are often accompanied by factors such as occlusion, rotation, and noise. These factors make it challenging to apply existing point cloud classification algorithms in real industrial scenarios. Currently, most studies enhance model robustness from the perspective of neural network structure. However, researchers have found that simply adjusting the neural network structure has proven insufficient in addressing the decline in accuracy caused by data corruption. In this article, we use local feature descriptors as a preprocessing method to extract features from point cloud data and propose a new neural network architecture aligned with these local features, effectively enhancing performance even in extreme cases of data corruption. In addition, we conducted data augmentation to the 10 intentionally selected categories in ModelNet40. Finally, we conducted multiple experiments, including testing the robustness of the model to occlusion and coordinate transformation and then comparing the model with existing SOTA models. Furthermore, in actual scene experiments, we used depth cameras to capture objects and input the obtained data into the established model. The experimental results show that our model outperforms existing popular algorithms when dealing with corrupted point cloud data. Even when the input point cloud data are affected by occlusion or coordinate transformation, our proposed model can maintain high accuracy. This suggests that our method can alleviate the problem of decreased model accuracy caused by the aforementioned factors.

## 1. Introduction

The problem of 3D scene perception is a very important computer vision task, with wide applications in many fields, such as remote sensing [1], security monitoring [2,3], face recognition [4], and autonomous driving [5,6]. In these practical application scenarios, point cloud data, as a very popular data type, contain rich data information and are easy to use. Therefore, especially in object classification tasks, point cloud data have great application value.

In the past, people mainly used traditional computer vision and image processing techniques to process point cloud data. Object recognition can be achieved by using features (3D coordinates, normal feature descriptors) of points with classical machine learning methods such as SVM and Random Forest. These methods combine local feature descriptors of point cloud data to assist traditional machine learning methods in object recognition tasks. This series of methods extract crucial point cloud information by compressing a large amount of point cloud data into local features. However, due to the difficulty of traditional machine learning algorithms in adapting to training with large amounts of data, the application of 3D point clouds in object classification tasks is still limited.

In recent years, deep learning has promoted the quality and accuracy of data processing. In the field of point cloud recognition, researchers have also utilized convolutional neural networks (CNNs) in 2D image classification [7,8]. At the same time, deep neural networks have also welcomed the 3D point cloud data form into the field of object classification. Since deep learning had been introduced into point cloud data processing, many excellent point cloud classification algorithms have been proposed [9,10,11,12]. These models employ sophisticated methods to address the issue of disorder in point clouds and achieved SOTA results on public datasets.

**Figure 1:**
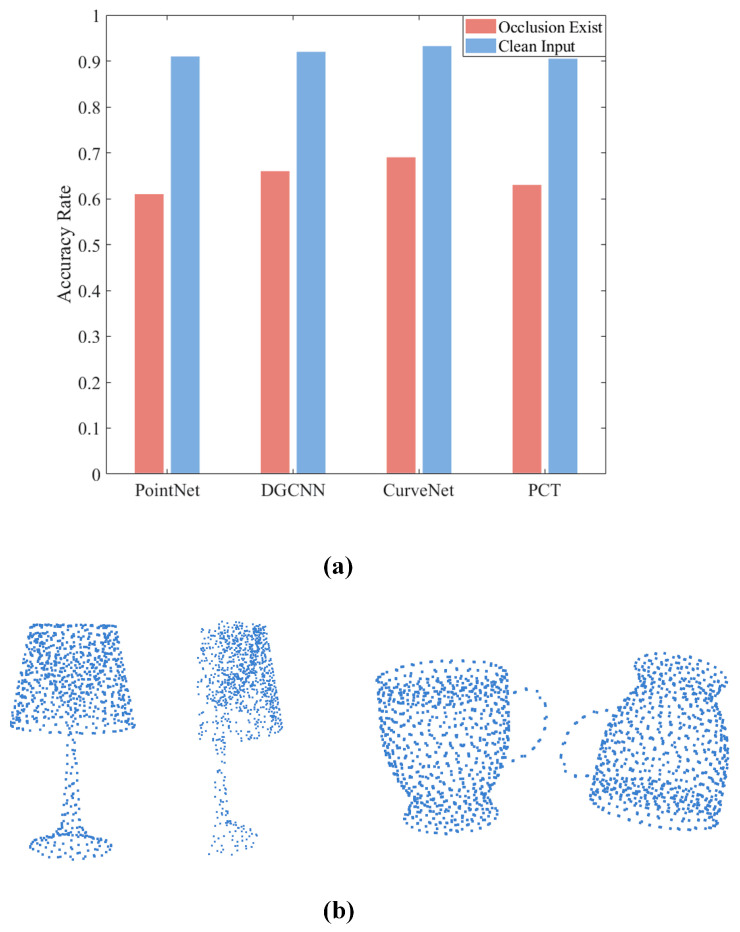
(**a**) SOTA neural network models perform well on clean point cloud data, but their testing accuracy decreases with real scene occlusion. (**b**) Examples of clean point cloud with corrupted point cloud: occlusion (left) and transformation (right).

However, these existing models still have some limitations. As we can see in Figure 1, while the aforementioned SOTA neural network models perform well on clean point cloud data, their testing accuracy decreases sharply when the point cloud data are corrupted [13]. These corruptions (noise, occlusion, transformation) are very common in actual industrial scenarios. Therefore, it is necessary to improve the performance of 3D point cloud classification models in extreme situations.

In response to the point cloud corruption issues, there is a certain research foundation for point cloud occlusion: Garcia-Garcia et al. [14] have studied the performance of 3D convolutional neural networks based on spatial voxels and CNN under occlusion conditions. Zhang et al. [15] modified the SOTA neural network based on latent features to improve its performance under occlusion conditions. M. A. Uy et al. [16] established ScanObjectNN to evaluate the performance of point cloud classification under the condition of point cloud data corruption in the real world. M. Y. Levi et al. [17] proposed using neural networks to extract key points to improve model robustness. H. Liu et al. proposed PointGuard [18], which downsamples point clouds and enhances neural network robustness through data augmentation. L. Zhou et al. proposed geometry-aware self-training (GAST) [19] for unsupervised domain adaptation of object point cloud classification. PointSetVoting [20] introduced a voting mechanism into the point cloud classification neural network to improve its robustness. In addition, researchers have combined point cloud completion models [21] with point cloud classification models to complete partial point cloud classification work.

Most studies aim to enhance model robustness from the perspective of neural network architecture. These solutions can enhance the model’s robustness to a certain degree. However, when the data are severely corrupted, even these robust models struggle with classification tasks. That is because the performance of neural networks largely depends on the input data, which means that optimizing solely from the perspective of neural network structure is insufficient. We believe that, in addition to changing the structure of neural networks, feasible solutions should also be provided from the perspective of input data. Point cloud data still need a series of preprocessing to reduce the impact of data corruptions.

At present, the data preprocessing methods of existing point cloud classification neural networks are relatively simple. Based on different neural networks, those methods can be divided into three categories:

- The first category is 2D convolutional neural networks based on multi-view [22,23,24], where the data preprocessing process involves converting 3D point cloud data into multi-view 2D images.
- The second type is convolutional neural networks based on 3D spatial voxels [25,26]. The data preprocessing process of such neural networks involves extracting information about the distribution patterns of points in the global space.
- The third type is neural networks based on 3D point cloud coordinates [12,27,28]. The data preprocessing process for such neural networks is the simplest, requiring only decentralization and normalization.

However, the data preprocessing methods currently used by neural networks make it difficult to mitigate the impact of damaged point cloud data. This is because the data preprocessing process of existing methods aims to preserve the original information of point cloud data. When point cloud data are damaged, the original data will be directly negatively affected to almost the same extent. We need a new data preprocessing method that can extract more undamaged information or features from point cloud data, in order to mitigate this negative impact.

Using point cloud local feature descriptors methods to extract undamaged features might yield better results. That is because local feature descriptors have very good properties, for example, FPFH [29] has fast computing speed and high real-time value, whereas spin image [30] has high robustness to occlusion. Introducing these local feature descriptors as preprocessing methods is expected to alleviate the decrease in model accuracy caused by the aforementioned problems to a certain extent. This will enhance the robustness of point cloud classification models to real scene corruptions.

In this article, we first examined the performance of point cloud recognition neural networks in extreme cases where point cloud data have different degrees of corruption. Then, we used local feature descriptors to extract point cloud data features. In addition, we built a new neural network structure in order to match the input local features and to enhance its performance in extreme cases of point cloud data destruction. After that, we conducted several experiments to test our method. Lastly, we conducted an ablation study to demonstrate the effectiveness of our methods.

Our research work will provide some references for practical applications and inspire new ideas for further research, thereby contributing to significant advancements in the field of point cloud classification, particularly for data with occlusion and other corruptions in real scenes.

## 2. Related Works: Local Feature Descriptors

Local feature descriptors [29,31,32,33] are widely used in the field of point cloud registration [34,35] and segmentation [36]; their application in point cloud classification tasks is less common. This section will provide a brief overview of commonly used point cloud local feature descriptors, followed by an introduction of the specific descriptors employed in this article.

### 2.1. 3DSC

The descriptor 3D Shape Context (3DSC) [37] performs a localized statistical analysis around each point. This offers significant advantages in terms of robustness to noise and random point distributions. Within the local spherical space representation, 3DSC exhibits lower sensitivity to random perturbations of individual points, granting it certain advantages over general curvature features.

### 2.2. NARF

Normal Aligned Radial Feature (NARF) [38] is an algorithm used to extract key feature points with object boundaries. The extracted feature points should be located in a stable area on the surface to ensure robustness in the estimation of normals, and there should be significant changes in the normals of neighboring points. Ideally, the extracted points should be located close to the object’s edges, so that most of the same feature points can be extracted from different viewpoints.

### 2.3. Spin Image

**Figure 2:**
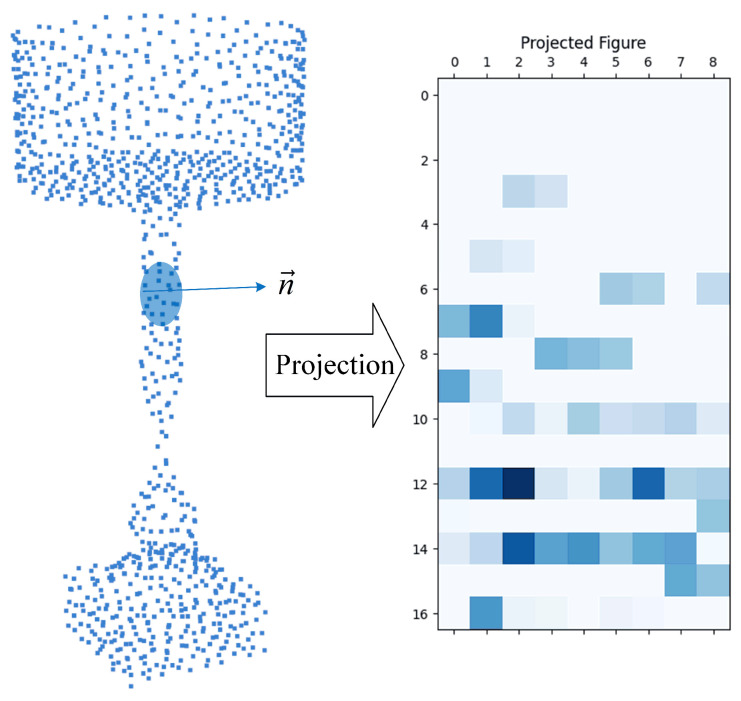
Visualization for spin image.

The steps of generating spin image features are as follows: First, an oriented point is defined as a point and its surrounding normal. Second, a cylindrical coordinate system is generated with the oriented point as the axis. Third, the three-dimensional point cloud inside the cylinder is projected onto a two-dimensional spin image. This process can be visualized as a spin image rotating 360 degrees around the normal vector $\vec{n}$. The points scanned by the spin image in three-dimensional space will be projected onto the 2D surface of the spin image. This process can be described as the following equations:

(1) $$S_{o}:\;R^{3}\to R^{2}$$

(2) $$\left(\alpha ,\beta \right)=(\sqrt{∥P-{Q∥}^{2}-{\left(\vec{n}\cdot \left(P-Q\right)\right)}^{2}},\vec{n}\cdot \left(P-Q\right))$$

where *n* represents the unit normal vector at point *P*; *Q* represents another vertex on the 3D mesh near *P*; and (α,β) represents the new coordinates of point *Q*. The new coordinate system will be divided into multiple grid cells. The strength of each grid is calculated based on the different points that fall into each grid in the spin image. In order to reduce sensitivity to position, reduce noise impact, and increase stability, Johnson et al. [30] used bilinear interpolation to distribute a point across 4 pixels. The main function of interpolation is to estimate the eigenvalues of non-discrete points on the surface of an object. It ensures the continuity and accuracy of descriptors across the entire surface of the object. The above process can be visualized as Figure 2.

We use the following analysis on several types of feature descriptors mentioned above to select one for our study. First, 3DSC uses the distribution characteristics of points in 3D space as descriptors, regarding the issues of rotation and occlusion. Moreover, due to the lack of a local coordinate system on the feature points of 3DSC, it may generate multiple feature descriptors [39]. As a result, it is difficult to achieve good preprocessing effects. For NARF, when corruption exists in point cloud data, it will lose some key points. As a result, NARF is more used for keypoint extraction rather than partial point cloud recognition [40]. Compared to the first two feature descriptors, spin image can eliminate some of the effects caused by missing point cloud data during the generation of 2D-rotated images [39], and it also has rotational invariance. Therefore, based on the above analysis, we will use spin image as the preprocessing tool for point cloud data.

## 3. Methods

### 3.1. Data Preprocessing Through Spin Image (SI)

For 3D models, uniform Poisson sampling is used to ensure that the sampling points are not too dense and generate a sparse sampling point cloud. This is performed in order to prevent individual positions from being extremely large in the input point cloud feature vector, which is beneficial for the neural network to learn the point cloud features. After uniform sampling, we input point cloud data (PCD) format point cloud data and use Point Cloud Library (PCL) [41] to generate spin image features.

**Figure 3:**
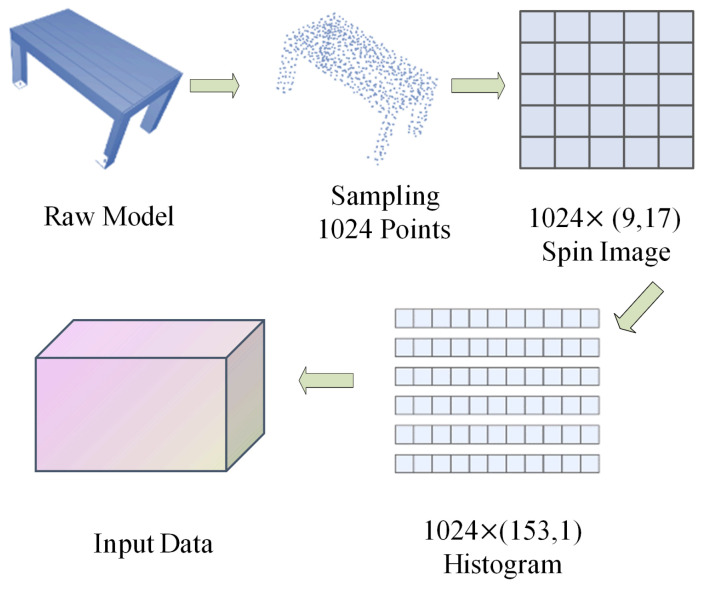
Sampling, SI feature creation.

In the spin image feature used, all uniformly sampled points will form a pair of points. For example, if 1024 points are uniformly sampled, we will obtain 1024 oriented points, resulting in 1024 rotated projection maps. We used 17 × 9 grid cells and normalized and vectorized them to obtain a frequency distribution histogram of 1024 × 153. In this way, not only are the data enhanced (obtaining histograms of output from multiple point pairs), but the features are also normalized, which is beneficial for the later learning of neural networks. The specific operation is shown in Algorithm 1 and Figure 3.
**Algorithm 1** SI Feature Creation Based on CAD Models.**Input:** Clean CAD models in OFF format**Output:** Neural network input tensors1:**for** each clean CAD *i* **do**2:   use Poisson disk sampling to obtain a point cloud $P_{i}$ of 1024 points;3:   **for** each point $p_{ij}\in P_{i}$ **do**4:     create a 2D basis $\left(p_{ij},\vec{n_{ij}}\right)$;5:     calculate the coordinates $(\alpha_{ik},\beta_{ik}),k\neq j$ of other points $p_{ik}$ in the new 2D coordinate system;6:     use bilinear interpolation to distribute a point across 4 pixels and create a (9,17) SI image;7:   **end for**8:   convert the obtained 1024×(9,17) SI images into an 1024×(153,1) SI histogram vectors;9:**end for**10:combine all 1024×(153,1) SI histogram vectors into tensors for neural network input;

### 3.2. Neural Network

#### 3.2.1. Inception Module

In the neural network, we first adopted the Inception network structure [42] to generate fused features. The Inception network can process the input single feature using different sizes of convolutional kernels. Before fully constructing this neural network, we attempted different convolutional kernel sizes. The experimental results showed that different kernel sizes would affect the performance of the neural network to an extent. To eliminate this influence, we used the Inception module and added convolution kernels of various categories from large to small. In the last part of this paper, we will explain the influence of convolutional kernels in the ablation study. By using convolution kernels of different sizes at the same level, the Inception module can simultaneously capture features of different scales, thereby improving the network’s recognition ability for objects of different sizes. This helps to improve the generalization ability and performance of the model. The Inception module has had a positive impact on the development of neural networks, improving their feature extraction capabilities and enabling them to better adapt to different tasks and application scenarios.

In our proposed network, the Inception module uses several types of convolution kernels with several sizes (we used 3 kinds of kernels A, B, and C for experiments; A is the largest one while C is the smallest one) to convolve the original size of (153, 1024) spin image features. The number of filters n within each kernel can be adjusted based on specific requirements. After the original point cloud feature data pass through the Inception module, the output will be a fused feature with a size of (n, 1024, 13).

#### 3.2.2. Attention Module (Squeeze-and-Excitation Blocks)

After using the Inception module to output fused features, if we directly input them to the final multilayer perceptron and perform pooling, the training results will be unsatisfactory and will produce severe overfitting. This is because the data we input ares augmented, and the regularity of the augmented data is poor. Simply using a linear layer to process the output features is incomplete. As a result, we added an attention module [43], which consists of a pooling layer and a fully connected layer. The first pooling layer is calculated as:

(3) $$z_{c}=\frac{1}{H\times W}\sum_{i=1}^{H}\sum_{j=1}^{W}u_{c}\left(i,j\right)$$

The dimension of the fully connected layer decreases first and then increases, aiming to extract the weights of each channel. The linear hidden layer can be described as:

(4) s=σ(W2ReLU(W1z))

Finally, the weights are multiplied to the corresponding channels:

(5) $$U_{c}=\left(1-\gamma \right)U+\gamma s⊗U$$

For each matrix element, the weights of the attention mechanism can be expressed as:

(6) $$u_{c}=\left(1-\gamma \right)u+\gamma s_{c}u$$

These weights help mitigate the influence of data irregularities to a certain extent. In general, attention mechanisms will extract features that are helpful for classification, filter out noisy features, and improve the performance of neural networks. This hyperparameter γ here controls the weight of the attention mechanism’s influence on the neural networks. We are going to use the hyperparameter later in the ablation study, which will give us an indication of the effect of the attention module in our neural network.

#### 3.2.3. Pooling Comparison Under Extreme Data Condition

**Figure 4:**
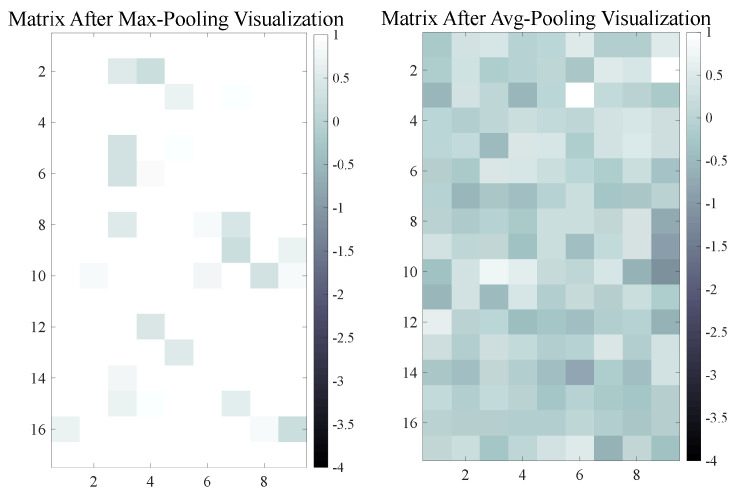
Comparison under data corruption. Max pooling (**left**): a large amount of irregular data. Average pooling (**right**): effective data can be maintained.

**Figure 5:**
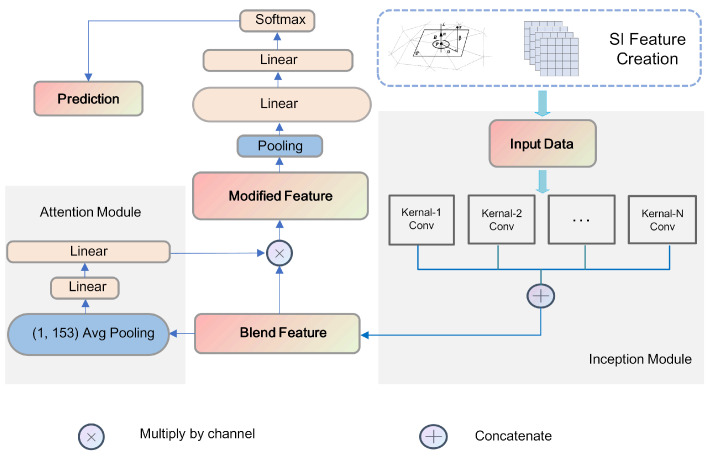
Neural network for SI features.

In the last section, we have not adopted the recently popular max pooling. Using max pooling can be problematic when the point cloud has defects. In such cases, a single large value caused by the defect might be maximized. In this way, using maximum pooling will result in this defect masking other residual features, i.e., important residual features that remain unaffected by the defect (neural networks often rely on these features for accurate classification). On the contrary, we adopted average pooling. For point clouds with minimal damage, average pooling preserves most residual features while minimizing the influence of extreme values caused by defects [44]. On average, neural networks can still make more rational judgments, as we can see by the result in the ablation study section. We conducted a small experiment to explain the above conclusion: we used four matrices, two of which contained normal and undistorted data, and the other two contained maxima (distorted). We processed a normal matrix with a distorted matrix using max pooling and average pooling separately. The final output is two matrices, as shown in Figure 4: the white area is highly irregular data (caused by distorted maxima features), while the dark area is valid useful data. From the results shown in Figure 4, it can be further speculated that when there is damage in the point cloud, the output data of the spin image feature F (with a dimension of (m,H,W), where m is the number of points) may have some local maxima in the $f_{i}$(H,W) rotation feature map obtained by some oriented points, while the rest of the area is small. When F(m,H,W) has multiple sub-matrices $f_{i}$ with local maxima, using max pooling will highlight these extreme features, resulting in some irregular features being amplified and outputted at the end. In contrast, if we use average pooling, these extreme features will be diluted, reducing their influence. The average pooling process can be described by the following equation and figure:

(7) $$\left\{\begin{array}{c} \;F=[f_{1},f_{2},f_{3},\ldots ,f_{m}]\\ \;O_{\mathrm{avg}}=\frac{1}{m}\sum_{i=1}^{m}f_{i}\\ \;O_{\max}=\max\left\{f_{1},f_{2},\ldots ,f_{m}\right\} \end{array}\right.$$

where F represents the input spin image feature tensor, and F is composed of input matrix $f_{i}$. $O_{\mathrm{avg}}$ and $O_{\max}$ represent average pooling and max pooling output, respectively. Figure 4 illustrates the respective results of two pooling processes:

The neural network are shown in Figure 5.

#### 3.2.4. Data Augmentation for Training

**Figure 6:**
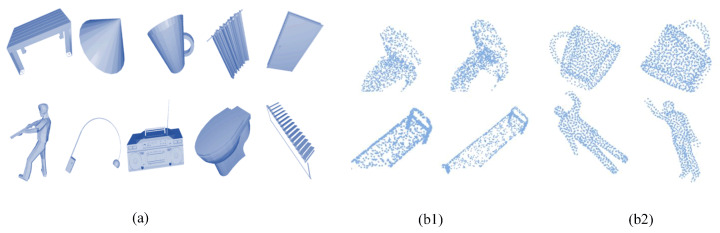
(**a**): Clean model of selected datasets. (**b1**): Corrupted point cloud with occlusion. (**b2**): Corrupted point cloud with transformation.

We created a dataset and used a subset of ModelNet40 to manipulate the point cloud. This subset contains enough data, which are divided into 10 categories (Figure 6a). We have created ModelNet10_Seg (Figure 6(b1)) and ModelNet10_Rot (Figure 6(b2)) for learning and testing, representing random segmentation (simulating occlusion effects) and random rotation (simulating rotation effects) for 10 types of point cloud data, respectively. Among them, ModelNet10_Seg also includes damaged point clouds with different occlusion rates (10%, 20%, 30%, 40%). These point cloud data are all standard with 1024 points.

The segmented data are created as following:

Firstly, using the Open3D library, the off-format files of the dataset were converted into PCD format through Poisson disk sampling. The numbers of sampling points were 1138, 1280, 1463, and 1707, respectively. Then, a portion of the points was randomly masked until the number of points decreased to 1024. This resulted in point cloud files with occlusion rates of 10%, 20%, 30%, and 40%, respectively.

**Figure 7:**
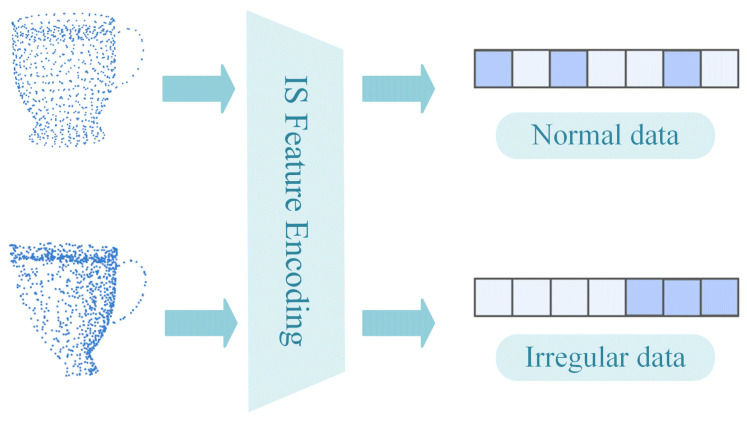
When there is an occlusion in point cloud data, the input data obtained from SI feature extraction will exhibit irregularities.

As shown in Figure 7, when the point cloud data contain occlusions, the distribution of partial point clouds is different from that of the complete point cloud, that is, the distribution of partial point clouds is irregular. Due to the irregular distribution of point clouds, the feature weights extracted by the local feature descriptors will exhibit significant differences, that is, the input data are irregular. As we mentioned before, data irregularity can be reduced to some extent by average pooling and attention mechanisms containing appropriate weights.

These datasets can be directly input into the feature extraction code, extracting the aforementioned feature vectors, and then inputting these vectors into the neural network. In addition, we intentionally extracted some models from ModelNet40 that are highly sensitive to occlusion, for example, lamps and toilets. In their complete state, they are easier to recognize, but in the presence of occlusion, their features become very indistinct. At the same time, we also added some objects that are difficult to classify by neural networks, such as doors and curtains. In the presence of occlusion, these two models will also become difficult to distinguish. In this way, the difficulty of deep neural networks for point cloud recognition tasks will also greatly increase.

Finally, for the complete point cloud data of each category, we added randomly segmented and randomly rotated point cloud data. To prevent the neural network from learning more extreme features, which have extreme specificity and may lead to a decrease in accuracy under low occlusion input or clean input, we only selected a portion for data augmentation. We mainly focused on data with low occlusion rates and added a small amount of corrupted data for training datasets. It is more beneficial to enhance the robustness of neural networks while maintaining high accuracy on low occlusion input.

## 4. Experiments

Our experiments are implemented using PCL [41] and Pytorch 2.0.1 on RTX 3060 GPU (NVIDIA Corporation, Santa Clara, CA, USA). Some important training hyperparameters and some basic modules of neural networks are listed in Table 1 and Table 2, respectively. We selected the best results during the training process. Afterward, we will use these training models for specific analysis.

### 4.1. Model Training

**Figure 8:**
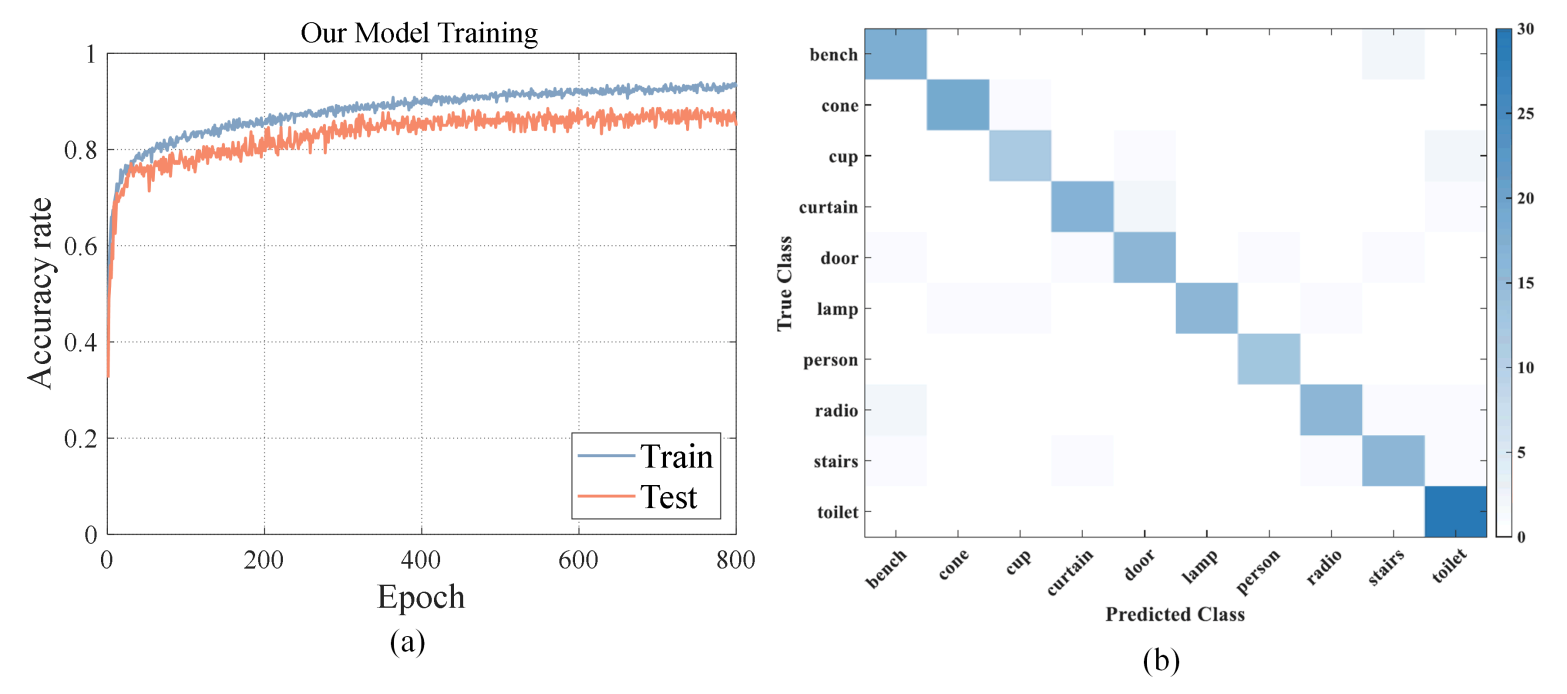
Training results: (**a**) Training epoch plots. (**b**) Confusion matrix of our model. Curtain and door are categories that are easily confused.

We trained our model on the augmented dataset constructed above, along with some existing SOTA models and typical robust point cloud classification models. The training results of our model are shown in (Figure 8a,b). We selected multiple models with good performance after the model accuracy stabilized. For other models, the best results obtained from training are shown in the first column of Table 3. PointNet’s [9] test accuracy can reach 91%. The accuracy of our proposed model is between 85% and 91%. The accuracy of DGCNN [10] can reach 92%, and the accuracy of CurveNet [12] can reach 91%. In addition, the robust point cloud classification neural network (Refocusing [17] with PointNet/DGCNN and PointSetVoting [20]) has relatively high overall stability and a certain degree of robustness.

We tested the good results obtained from training with clean point cloud and 90% occlusion point cloud data sets and created a confusion matrix. It can be seen in Figure 8b that door–curtain is a category pair that is easily confused by the neural network. In addition, the neural network performed poorly on the stairs and lamp categories, with many classification errors occurring. In this way, the model training results verify the particularity of the construction of the aforementioned dataset.

### 4.2. Experiments for Occlusion

**Figure 9:**
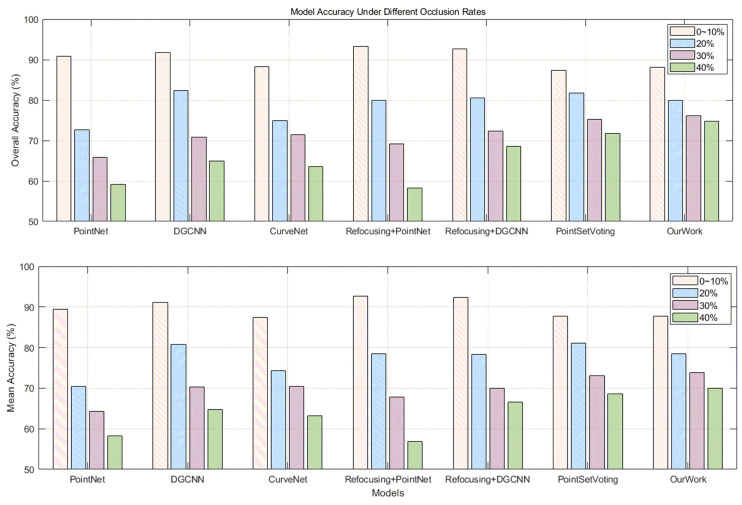
Testing results: PointNet (+Refocusing), DGCNN(+Refocusing), and CurveNet—as the occlusion rate increases to about 27%, MA and OA of four models will decrease sharply. OurWork—when the occlusion rate increases from 30% to 40%, the overall and mean accuracy rates remain relatively stable.

After we trained our proposed neural network using the augmented dataset (including the complete point cloud and a portion of the point cloud data with an occlusion rate of 10%), we used the remaining point cloud data (with an occlusion rate of 20% to 40%) containing occluded point cloud data for testing. According to Figure 9 and Table 3, we can see that as the occlusion rate increases to about 27%, the accuracy of existing models will decrease to some extent, but our model decrease rate is relatively small. As shown in the Table 3 and Table 4, the accuracy is always higher than 70%.

On the other hand, although models like PointNet perform well in complete point cloud recognition tasks, their accuracy can significantly decrease when there are defects and damage in the point cloud data (when the occlusion rate increases by 10%, the model’s prediction accuracy decreases by about 6% to 7%). Notably, when the occlusion rate of point cloud data rises to 35%, the prediction accuracy will drop very low. The occlusion rate of some of the point cloud data we obtain in reality is generally not lower than 30%, which poses a challenge to all point cloud recognition networks.

**Figure 10:**
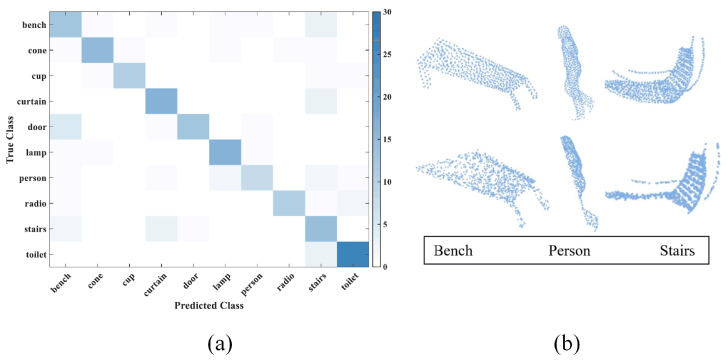
(**a**): Confusion matrix of our model. Door–bench is a category that is easily confused. Complex models are difficult to recognize, such as person–stair. (**b**): Confusing categories: door–bench; person–stair.

Finally, we have analyzed the shortcomings of the models above. In addition to the confusing categories curtain and door, which we intentionally selected to have high error rates when constructing the dataset, we list other categories with high model recognition error rates (Figure 10). As shown in the figure, when the occlusion rate is high, we selected three models with higher error rates from the above 10 models, namely bench, person, and stairs. These models each have their own characteristics: when the camera shoots the bench model from the top, its surface can easily confuse the model with door. In addition, for person and stairs, the surface features of these two models are complex. Once the occlusion rate increases, the spin image feature descriptor loses more features, which will greatly affect the performance of the neural network.

### 4.3. Experiment for Transformation

**Figure 11:**
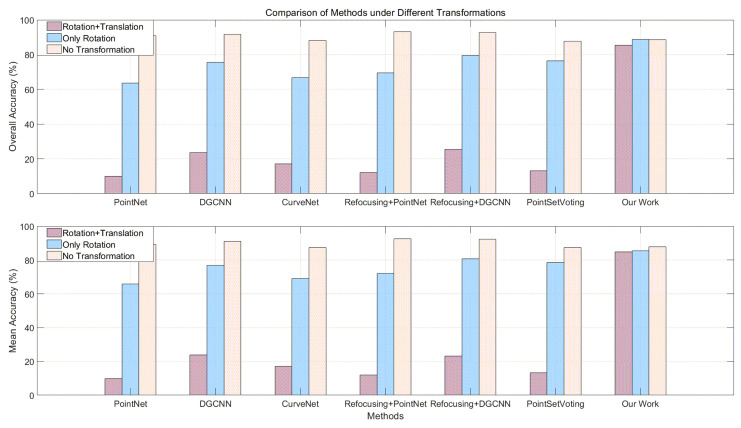
Testing results: (1) During the process of random rotation, SOTA models and robust point cloud models will be more or less affected. Our proposed new model performs relatively better. (2) With translation, SOTA models and robust point cloud models cannot recognize the input data correctly. Data must be decentralized and normalized.

Our evaluation experiment on rotation is conducted on the trained models, and the results are shown in the Table 5 and Table 6. We evaluated these models using the created ModelNet10_Rot. In this experiment, we found that models like PointNet have a certain degree of robustness to rotation. However, during the process of random rotation, these models will be more or less affected. In this experiment, for instance, the accuracy of the PointNet model decreased by about 26%. Relatively, our proposed new model performs better on data with random rotations. Even when the data are randomly rotated, its classification accuracy increases by about 0.2% (obviously, this is highly likely caused by random errors). Therefore, we can consider that our model has strong robustness to rotation. When there is a translation in coordinate transformation, the center of the point cloud will change (Figure 11). If the point cloud data are transformed before input into the SOTA models, it will not be able to complete the recognition task correctly. Using local feature descriptors can avoid this problem.

### 4.4. Real Scene Experiment

**Figure 12:**
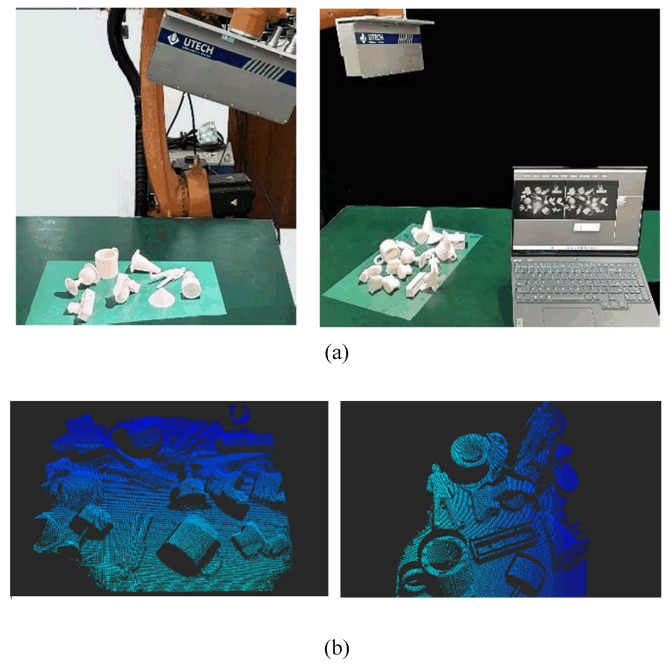
(**a**): Real scene experiment. (**b**): Obtained depth image.

**Figure 13:**
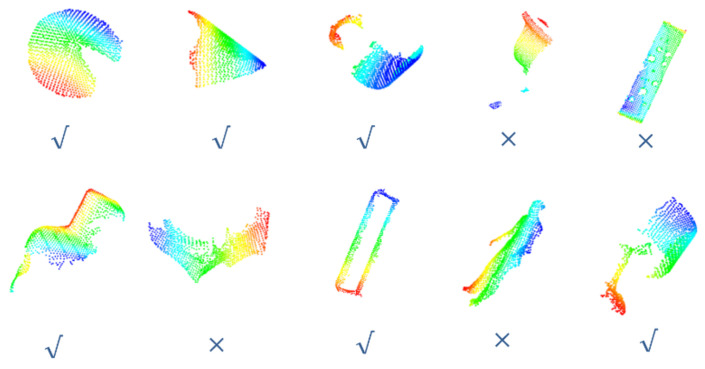
Testing result of real scene experiments.

In order to compare the point cloud data obtained from actual shooting with publicly available datasets online, we conducted this real-scene experiment. Firstly, we used a 3D printer to reconstruct several 3D models from the publicly available datasets. Afterwards, as shown in the Figure 12, we used a depth camera to capture the printed 3D model. We try to simulate the actual scene as much as possible when placing 3D models, such as the cluttered scene on the desktop. Finally, we segment the point cloud and input it into the aforementioned algorithm. When we use public datasets and simulate their occlusion, our focus is on the limited shooting angle of depth cameras, because depth cameras only shoot from a single perspective. We did not consider the interference between objects. In this experiment, we considered more factors, such as introducing not only the corruptions caused by the perspective of the depth camera and the rotations caused by the angle of the depth camera but also the occlusions between items and the rotation angles when placing items. From the results in the figure below, it can be seen that the actual scenario has brought more difficulties to the neural network. The accuracy of the model has decreased significantly, especially for models with more complex surfaces, such as toilets and persons (Figure 13), and easily confused models, such as benches. The model performs poorly on these models, but for models with relatively smooth surfaces, the model can still maintain strong robustness.

### 4.5. Ablation Study

We conducted four experiments for this part, in order to give an indication of the effect of the attention module in our neural network. The results of these experiments concluded that in this model, the importance of each module is graded as follows: first—pooling layer, second—convolutional kernel combination form, and third—attention mechanism.

#### 4.5.1. Ablation with Inception Module

We have adjusted different combinations of convolution kernels. As we can see in Table 7 and Table 8, the size of the convolution kernel can affect the performance of the neural network to some extent. However, in order to mitigate this effect, we can use the convolution results obtained from multiple convolution kernels during the adjustment of the network structure and combine them into a new feature. In this way, the neural network does indeed perform better on datasets with poor regularity.

#### 4.5.2. Ablation with Attention Module

The attention module plays an optimizing role in the proposed neural network. It can adjust the weights of the learned features of the neural network to a certain extent. In the attention mechanism, we change hyperparameter γ to adjust the impact of the attention mechanism on the entire neural network model. As can be seen from Table 9 and Table 10, the weight of the attention mechanism in the neural network is not necessarily the larger the better. For instance, when γ=1, indicating a relatively larger weight on the attention mechanism, the model’s performance is inferior to that of γ=0.5. However, it is observed that when γ=0, meaning the complete exclusion of the attention mechanism, the model’s performance is the least desirable. Whether utilized in point cloud classification tasks involving occlusions or in tasks involving coordinate-transformed point clouds, models devoid of attention mechanisms consistently exhibit lower accuracy compared to those incorporating attention mechanisms.

#### 4.5.3. Pooling Layer

After the Shared MLP of the model, there is a pooling layer. We trained the best model using max pooling and average pooling separately. The results are shown in Table 11 and Table 12. It can be observed that once max pooling is used, both the accuracy of partial point cloud recognition and the robustness of the model to transformation decreased significantly. This experiment shows that average pooling can perform better under extreme data conditions.

#### 4.5.4. Different Hyperparameters

Finally, we adjusted the hyperparameters. The first set of data in Table 13 can make the neural network perform better, while the rest of the hyperparameters, such as $\beta_{1}$ and $\beta_{2}$ in the Adam optimizer [45], have little impact on the performance of the neural network. Therefore, for the rest of the hyperparameters, we used the default values of during training.

#### 4.5.5. New Datasets Testing

**Figure 14:**
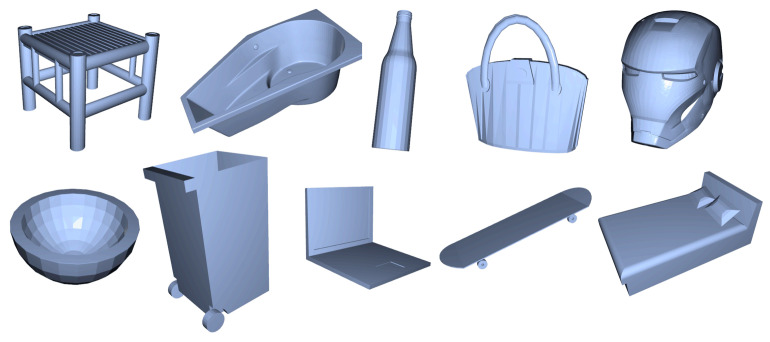
CAD Models of ShapeNet.

**Figure 15:**
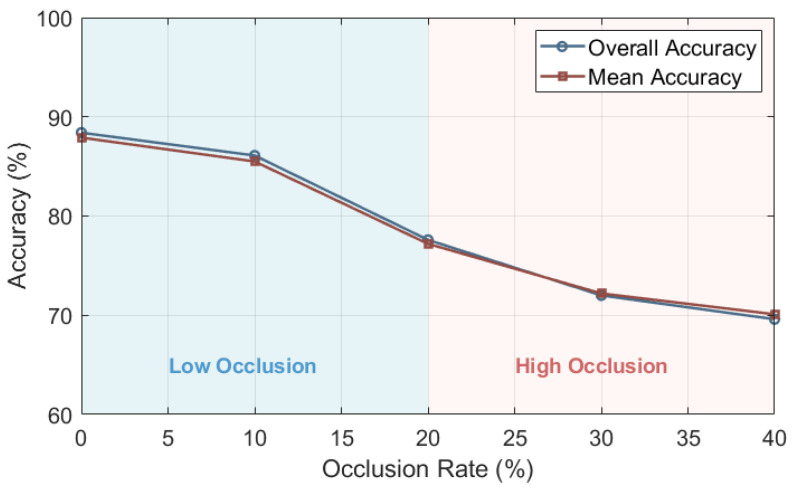
Testing results of ShapeNet with occlusion: our model can maintain strong robustness in the presence of occlusion in point cloud data, especially when the occlusion rate is high.

**Figure 16:**
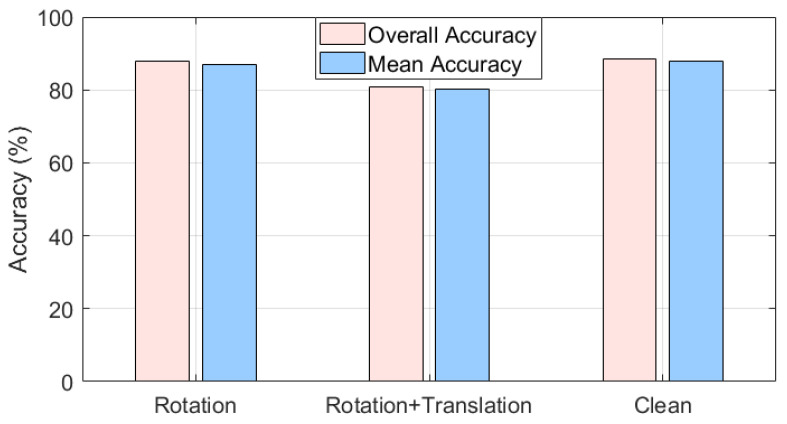
Testing results of ShapeNet with transformation: our model can maintain high robustness when there is coordinate transformation in the input point cloud data.

In this section, to examine the performance of the point cloud classification model proposed on different datasets. We trained our model on 10 categories of ShapeNet (Figure 14). The data preprocessing method and corresponding algorithms are as previously described. As shown in the Table 14, Figure 15 and Figure 16, our model has strong adaptability to different datasets, and can still maintain strong robustness against incomplete data in different datasets.

### 4.6. Discussion

Throughout the entire experimental process, we analyzed the effects of different occlusion rates and coordinate transformations on point cloud classification neural networks. It can be seen from the accuracy of the neural network model that point cloud classification with corruption is a very challenging task. It can be seen from the results that our model performs better compared to existing models. We can explain the reasons using the following three aspects:

- Feature descriptors play a role in alleviating incomplete data, and even in the presence of occlusion in point clouds, local feature descriptors can still extract effective local information and input it into neural networks.
- The average pooling process in neural networks also reduces the negative impact caused by local data failure.
- The attention module can effectively improve the stability of the model in extreme situations.

In addition, point cloud classification neural networks that use point cloud data directly as input may have an unsatisfying performance due to point cloud coordinate transformations. For example, although algorithms such as PointNet alleviate the impact of rotation to some extent, the recognition accuracy of these algorithms that use the original coordinates of the point cloud for recognition still significantly decreases. Moreover, when the point cloud data undergo translation and rotation, as shown in Figure 11 and Table 5, the performance of these algorithms becomes very unsatisfactory. We can only effectively work with these algorithms by re-transforming the point cloud model so that its centroid is located at the original position. Compared to the other three methods, our method utilizes local feature descriptors of point clouds. As a result, the data input to the neural network is almost unaffected by changes in point cloud coordinates, and after pooling processing, the disorder problem of points can be effectively solved. Therefore, compared to neural networks that directly input 3D point cloud coordinates, neural networks based on local features of point clouds have an advantage for identifying point cloud data that have undergone spatial transformations.

Finally, we conducted an ablation study with a focus on understanding the impact of neural network module adjustments on the performance of point cloud classification tasks. We can see that the pooling layer is the module that has the greatest impact on the performance of the classification neural network. We tend to choose average pooling to mitigate the impact of extreme data when data regularity is poor. The experimental results (Table 11 and Table 12) also confirmed this point. The attention mechanism has a relatively smaller impact on neural networks, but it can enhance the stability of neural network performance. This is because, during the training process of the model, we found that the larger the weight of attention mechanism parameters, the more stable the final accuracy of the model. Moreover, as we can see in Table 9 and Table 10, the model without the attention module is least satisfactory. Thus, we can make a conclusion that the attention mechanism can make the neural network more robust to corruption and assist the neural network in learning irregular datasets. The Inception module has a relatively significant impact on neural networks, and it can be concluded that when the number of convolutional kernels is not too large and the training time and computational requirements are not high, the richer the number of different kernels, the better the model performance.

## 5. Conclusions

In this paper, we propose a point cloud classification neural network based on point cloud local feature descriptors, which effectively improves the robustness of the point cloud classification model to occlusions and coordinate transformations and thus promotes the application of point cloud classification in real industrial scenarios. To achieve this, local feature descriptors are first employed to extract point cloud features as a robust preprocessing method. In addition, a new constructed robust point cloud classification neural network is adapted to point cloud feature descriptors, thereby further improving the performance of the model in corrupted point cloud data. Extensive testing and real-scene experiments based on ModelNet have shown that our model outperforms existing SOTA models and other robust point cloud classification models.

However, the proposed method still has shortcomings. In practical experiments, simple models still exhibit misidentification. This is because, in actual scenarios, there may still be noise in the sampled point cloud, as well as factors such as overlap between models. In future research, we will consider combining multiple robust feature descriptors and further adjusting the neural network for better practical application results. 

## Figures and Tables

**Table 1:** Hyperparameters in the neural networks.

Symbol	Meaning	Value
α	learning rate	0.00015 (default)
λ	weight decay	0.00300 (default)
γ	weight of attention module	0 <γ< 1, for tuning
A,B,C	3 different kernel sizes	A (17,1); B (51,1); C (153,1)
*p*	drop out probability	0.60 (default)

**Table 2:** Other modules in the neural network.

Module	Method
Activate Function	ReLU
Loss Function	CrossEntropyLoss
Optimizer	Adam [45]

**Table 3:** Overall accuracy (OA) comparison with occlusion.

	0∼10% ^3^	20%	30%	40%
PointNet ^1^	90.9	72.7	65.9	59.1
DGCNN ^1^	91.8	**82.3**	70.9	65.0
CurveNet ^1^	88.2	75.0	71.4	63.6
Refocusing + PointNet ^2^	**93.2**	80.0	69.1	58.2
Refocusing + DGCNN ^2^	92.7	80.5	72.3	68.6
PointSetVoting ^2^	87.3	81.8	75.3	71.8
OurWork	88.1	80.0	**76.2**	**74.7**

^1^ Existing SOTA models on clean datasets. ^2^ Robust point cloud classification models proposed in recent years. ^3^ We trained these models on an augmented dataset. It contains a portion of low occlusion rate point cloud data, with occlusion rates ranging from 0 to 10. Bold data shows the best performance under the same data conditions.

**Table 4:** Mean accuracy (MA) comparison with occlusion.

	0∼10%	20%	30%	40%
PointNet	89.4	70.5	64.3	58.2
DGCNN	91.2	80.8	70.3	64.7
CurveNet	87.4	74.3	70.5	63.2
Refocusing + PointNet	**92.6**	78.4	67.8	56.9
Refocusing + DGCNN	92.3	78.3	70.0	66.6
PointSetVoting	87.7	**81.1**	73.0	68.6
OurWork	87.8	78.5	**73.8**	**70.0**

**Table 5:** OA comparison with transformation.

	Rotation + Translation	Only Rotation	No Transformation
PointNet	10.0	63.6	90.9
DGCNN	23.5	75.5	91.8
CurveNet	17.0	66.8	88.2
Refocusing+PointNet	12.0	69.6	**93.2**
Refocusing+DGCNN	25.5	79.6	92.7
PointSetVoting	13.0	76.4	87.7
Our Work	**85.5**	**88.7**	88.5

**Table 6:** MA comparison with transformation.

	Rotation + Translation	Only Rotation	No Transformation
PointNet	9.8	65.8	89.4
DGCNN	23.8	76.9	91.2
CurveNet	17.2	69.2	87.4
Refocusing+PointNet	12.0	72.0	92.6
Refocusing+DGCNN	23.2	80.8	92.3
PointSetVoting	13.2	78.6	87.3
Our Work	**84.7**	**85.7**	87.8

**Table 7:** Inception ablation study with occlusion.

Kernels	OA (0–10%)	OA (20%)	OA (30%)	OA (40%)
A	85.3	76.2	68.3	61.7
AB	86.1	74.6	71.9	70.5
ABC	88.5	82.0	76.2	77.8

**Table 8:** Inception ablation study with transformation.

Kernels	OA (Rotation)	OA (Rotation + Translation)
A	83.0	81.1
AB	85.8	82.9
ABC	88.7	85.5

**Table 9:** Attention ablation study with occlusion.

Parameter γ	OA (0∼10%)	OA (20%)	OA (30%)	OA (40%)
1	88.1	80.0	76.2	74.7
0	86.4	74.6	74.5	72.3
0.2	88.1	77.3	75.7	73.2
0.5	88.5	82.0	76.2	77.8
0.8	89.9	78.4	75.1	75.3

**Table 10:** Attention ablation study with transformation.

Parameter γ	OA (Rotation)	OA (Rotation + Translation)
1	86.2	83.9
0	85.6	83.3
0.2	88.7	85.5
0.5	88.7	85.5
0.8	88.2	85.0

**Table 11:** Pooling ablation study with occlusion.

Pooling Layer	OA (0∼10%)	OA (20%)	OA (30%)	OA (40%)
Max Pooling	76.5	58.7	57.7	51.9
Average Pooling	88.5	82.0	76.2	77.8

**Table 12:** Pooling ablation study with transformation.

Pooling Layer	OA (Rotation)	OA (Rotation + Transformation)
Max Pooling	77.8	79.0
Average Pooling	88.7	85.5

**Table 13:** Hyperparameters tuning.

*p*	α	λ	OA (0∼10%)
0.66	0.00011	0.00300	88.5
0.64	0.00015	0.00225	87.9
0.65	0.00018	0.00200	88.1

**Table 14:** Testing results under different data conditions on ShapeNet10.

Data Condition	OA	MA
0% Occlusion	88.4	87.9
10% Occlusion	86.1	85.5
20% Occlusion	77.6	77.2
30% Occlusion	72.0	72.2
40% Occlusion	69.6	70.1
Rotation	87.9	87.1
Rotation + Translation	80.8	80.3

## Data Availability

This study is an experimental analysis of a public dataset. The raw data supporting the conclusions of this article will be made available by the authors on request.

## Abbreviations

The following abbreviations are used in this manuscript:

SOTA	State Of The Art
SI	Spin Image
ReLU	Rectified Linear Unit
PCD	Point Cloud Data
PCL	Point Cloud Library
3DSC	3D Shape Context
NARF	Normal Aligned Radial Feature
FPFH	Fast Point Feature Histogram
CNN	Convolutional Neural Network
SVM	Support Vector Machine
OA	Overall Accuracy
